# West Texas Medical Association

**Published:** 1895-12

**Authors:** 


**West Texas Medical Association.**—This association held its 19th annual session in San Antonio, Thursday October 31st, with a full attendance. Some good papers were read and discussed. We hope to be able to give them in the next issue, or some of them. The polite secretary, Dr. J. T. Fitz Simon, sends us a brief report of the meeting, the president's address, and the names of the new officers.

President, Dr. B. E. Hadra, San Antonio.

First Vice-President, Dr. R. E. Moss, San Antonio.

Second Vice-President, Dr. A. Garwood, New Braunfels.

Secretary and Treasurer, Dr. J. T. Fitz Simon, San Antonio.

CENSORS.—Dr. H. A. Tutwiler, Flatonia; Dr. Frank Paschall, Dr. F. M. Hicks, Dr. B. F. Kingley, San Antonio; Dr. J. R. Evans, Devine.

Dr. H. A. Tutwiler, the retiring president, delivered an interesting address, taking a good deal of latitude, touching on numerous subjects of interest to doctors, paid a tribute to the memory of recently departed members—Cupples and Paulus—and closed with a strong plea for State care of epileptics.

We shall have more to say of this later. Association closed its meeting with a banquet, at which wine, wit and wisdom flowed with equal pace.

The West Texas Medical Association has an active membership of 48 good men and true, and keeps up its organization and interest, and never lets them flag.

---

**At the** last meeting of the Tri-State Medical Society (of Iowa, Illinois and Missouri) the following officers were elected:

President, Dr. Robt. H. Babcock, Chicago.

1st Vice-President, Dr. A. H. Cordier, Kansas City.

2d Vice-President, Dr. W. A. Todd, Chariton, Ia.

Treasurer, Dr. C. S. Chase, Waterloo, Ia.

Secretary, Dr. G. W. Cale, St. Louis.

The next meeting will be held in Chicago the first Tuesday, Wednesday and Thursday, in April, 1896.

